# Editorial: Crosstalk in tumor microenvironments: shaping early drug and immunotherapy strategies

**DOI:** 10.3389/fimmu.2025.1754096

**Published:** 2026-01-08

**Authors:** Guendalina Froechlich, Alessandro Arcucci, Emanuele Sasso

**Affiliations:** 1 Department of Molecular Medicine and Medical Biotechnology, University of Naples Federico II, Naples, Italy; 2 CEINGE—Biotecnologie Avanzate Franco Salvatore S.c.a.r.l., Naples, Italy; 3 Department of Public Health, University of Naples Federico II, Naples, Italy; 4 ImGen-T Srl, Naples, Italy

**Keywords:** TME, MAbs, tumor niche, prognostic factors, stroma cells

The tumor microenvironment (TME) is no longer considered as an inert bystander but rather as a complex and dynamic ecosystem that profoundly interacts with cancer cells directly affecting tumor evolution, therapeutic resistance, and immune evasion. Malignant cells coexist with stromal, immune and endothelial cell populations, as well as with extracellular matrix (ECM), generating an intricate system of reciprocal interactions that critically determine disease trajectory. With the advent of single-cell sequencing, spatial omics, and high-resolution imaging, the field has moved beyond binary simplistic classifications of pro-tumor versus anti-tumor components. These technologies now enable unprecedented insight into cellular heterogeneity, lineage evolution, spatial architecture, and communication circuits that shape cancer progression. The goal of this Research Topic, *“Crosstalk in Tumor Microenvironments: Shaping Early Drug and Immunotherapy Strategies”*, is to gather studies that deepen our understanding of TME-mediated mechanisms relevant to early-stage drug development and immunotherapy design.

## TME-driven biomarkers, omics signatures, and prognostic frameworks

A central theme emerging across multiple contributions is the use of omics-driven frameworks to decode stromal and immune heterogeneity and derive prognostic markers. In triple-negative breast cancer (TNBC), Li et al. leveraged single-cell RNA-seq dataset, integrating network algorithms and a machine-learning pipeline to identify stromal cell signatures. In particular, the authors identified how three stromal cell subpopulations (i.e., myofibroblastic cancer associated fibroblasts myCAF, vascular smooth muscle cells VSMCs and pericytes) are associated with immunosuppression and poor outcomes. Their nine-gene model not only stratified patient survival but also anticipated responsiveness to immunotherapy, underscoring how stromal-derived transcriptional programs condition therapeutic susceptibility paving the way for future precision medicine. Similarly, Sui et al. dissected esophageal squamous cell carcinoma at single-cell resolution, identifying malignant subpopulations enriched in apoptosis-related pathways and reconstructing a risk signature driven by CTSC. Their findings, supported by functional assays confirming CTSC’s role in proliferation, migration, and TME remodeling, highlight how malignant cell states and stromal interplay co-evolve to influence immunotherapy resistance. In a third study by Varricchio et al. the authors focused on Hodgkin lymphoma, revealing that FKBP51 expression within CD4^+^ tumor-infiltrating lymphocytes, more than in Hodgkin/Reed-Sternberg cells, was a critical determinant of clinical outcome. Their spatial mapping of TME architecture demonstrated how CD4^+^ rosettes and tumor-associated macrophages (TAMs) contribute to a protumoral niche. Together, these omics-driven studies emphasize that both stromal programs and immune cell spatial patterning function as powerful prognostic dimensions, emerging as integral components of disease classification.

In a comprehensive review, Wen et al. underscore that the nervous system actively tunes the TME. CNS-derived neurotransmitters and neuropeptides can alter immune cell behavior: for example, sympathetic norepinephrine can upregulate PD−1 on immune cells and bolster suppressive macrophages/MDSCs, damping antitumor immunity. Tumors also recruit nerve fibers via neurotrophic factors, reshaping local immunity. Targeting this neuro−immune crosstalk (e.g. with β-blockers) is promising, but redundant pathways and broad neural roles may complicate therapy.

Spitschak et al. reveal a cell-intrinsic loop where melanoma E2F1 drives autocrine IL−6 secretion. The IL−6/STAT3 axis amplifies E2F1 signals, promoting invasion and skewing T cells toward a Th2, immunosuppressive phenotype. Knocking down E2F1 flips the TME toward Th1 immunity, suggesting that disrupting such loops might reinvigorate antitumor responses. Although IL−6/STAT3 are druggable, systemic blockade risks side effects and tumor heterogeneity could limit benefit.

## Antibody and drugs-based remodeling of TME

A distinct cluster of contributions centered on antibody-based strategies targeting immune modulation. Rapuano Lembo et al. generated a panel of fully human anti-OX40 monoclonal antibodies that recognize distinct epitopes and exhibit differentiated biological behaviors. The authors dissected the biological activity of OX-40 targeting mAbs focusing on how different recognized epitope together with glycosylation states of OX-40 shape receptor engagement on T cells. Also the authors focused on the role of OX-40 in NK cells offering mechanistic insights relevant for next-generation co-stimulatory agonists. Combinations of their antibodies enhanced activation, and their non-overlap with clinically validated epitopes such as Rocatinlimab suggests therapeutic complementarity. Adding to the immunotherapy landscape, Jin et al. identify transcriptomic biomarkers predicting responsiveness to TAVO412, a trispecific EGFR/c-Met/VEGF-A antibody, underscoring the value of gene signatures in stratifying patients for multi-target biologics. On the clinical front, Song et al. provide a systematic assessment of dual PD-1/PD-L1 and CTLA-4 blockade in advanced colorectal cancer, showing modest but meaningful responses despite substantial toxicity. Finally, Lu et al. reports a compelling case of gastroesophageal junction hepatoid adenocarcinoma achieving major pathological response through sintilimab-based chemo-immunotherapy, highlighting the potential of PD-1 inhibition in aggressive, poorly characterized tumor entities.

In a distinct but for such aspects connected to immune checkpoints several studies explored tumor immunity through the lens of myeloid regulation and TAM-targeted approaches. Wang et al. provided a comprehensive review of Stanniocalcin-1 (STC1) as a phagocytosis checkpoint regulating macrophage activation, polarization, and antigen presentation. By summarizing data showing STC1’s involvement in EMT, immunosuppression, and impairment of “eat-me” molecules, Wang et al. underscored the emerging concept that manipulating macrophage-tumor interactions is a promising direction for immunotherapy development. These works, taken together, highlight how antibodies and myeloid-modulatory agents represent parallel paths to reprogram immune responses within the TME.

Dumut et al. report that a novel anti-cancer compound, namely disulfiram-derived copper complex (CuET) pharmacologically “ignites” the TME. In colorectal models it turned “cold” tumors hot, driving lymphocyte infiltration and boosting NK/T cell cytotoxicity. CuET upregulated NKG2D ligands on cancer cells, improving immune recognition. CuET transforms the colorectal cancer TME, sustaining NK and T cell cytotoxicity and refining tumor cell recognition through non-conventional activation via the NKG2D/NKG2DL axis. This raises the prospect of repurposing DSF/Cu to enhance immunotherapy. However, careful dosing and validation in diverse tumors are needed.

Multiple studies in this Research Topic delve into how specific stromal or immune components influence tumor progression and therapeutic failure. The crosstalk between stromal fibroblasts, immune cells, and malignant cells emerges repeatedly as a dominant theme. The findings across these articles converge on a consistent message: stromal cells are not passive scaffolds but dynamic regulators of cytokine networks, extracellular matrix remodeling, and metabolic rewiring, all of which dictate immune surveillance and drug penetration. Several articles highlight how intrinsic tumor pathways interface with the immune landscape to shape disease trajectory. These works underline a critical emerging concept: cancer cell–intrinsic vulnerabilities often become actionable only when contextualized within TME pressures, and immunotherapy effectiveness cannot be fully predicted without considering both compartments. Collectively, the research gathered in this Research Topic forms a coherent picture: TME complexity is not merely a challenge but an opportunity—one that can be harnessed to design therapies that exploit stromal vulnerabilities, alter immune cell behavior, and overcome tumor evasive strategies.

## Future directions

Looking ahead, several promising directions emerge from the collective insights of this Research Topic. First, integrating single-cell and spatial transcriptomics into early-stage drug development will be essential to map not only who is present in the TME but how cellular interactions evolve under therapeutic pressure. Second, stromal and macrophage-targeted therapies—highlighted by the STC1 axis and CAF-derived programs—represent a frontier for combination strategies designed to reshape immunologically “cold” tumors into responsive ones. Third, the diversity of OX40-targeting antibodies generated by Rapuano Lembo et al. exemplifies how epitope-specific engineering can unlock new layers of immune activation, suggesting that next-generation immunotherapies may rely on fine-tuned modulation rather than inhibition alone. Finally, the integration of machine learning-derived prognostic signatures into clinical workflows offers a path toward patient-specific immunotherapy design, ensuring that therapeutic strategies are aligned with the underlying ecology of each tumor.

